# Genetic Diversity and Population Structure of the Kustanay Horse Breed Based on 16 STR Markers

**DOI:** 10.3390/ani16213453

**Published:** 2026-11-01

**Authors:** Bakhit Baimenov, Alma Dossova, Stepan Khlyupko, Vitaly Raketsky, Aigul Tastemirova, Vadim Ulyanov, Tatyana Ulyanova

**Affiliations:** 1 Laboratory of Molecular-Genetic Research, Research Institute of Innovative Technologies, Akhmet Baitursynuly Kostanay Regional University, Kostanay 110000, Kazakhstan; bahytbajmenov@gmail.com (B.B.); dossova.alma95555@gmail.com (A.D.); 2 Kostanay Branch, Kazakh Scientific Research Veterinary Institute LLP, 94 Dulatov Street, Kostanay 110000, Kazakhstan; hlupkostepan@gmail.com (S.K.); vitali29071982@gmail.com (V.R.); 3 Republican Chamber for Pedigree Horse Breeds, 6/137 Korgalzhyn Highway, Astana 010000, Kazakhstan; amandykova3393@gmail.com; 4 Laboratory of Biotechnology and Diagnostics of Infectious Diseases, Testing Center, Zhangir Khan West Kazakhstan Agrarian and Technical University, 51 Zhangir Khan Street, Uralsk 090009, Kazakhstan

**Keywords:** Kustanay horse, microsatellite markers, genetic diversity, population structure, Kazakhstan

## Abstract

The Kustanay horse is a riding breed developed in Kazakhstan whose genetic diversity and differentiation remain insufficiently studied. We assessed genetic diversity and population structure using 16 autosomal STR loci from a 17-locus panel comprising 12 ISAG core markers. Of 765 genotyped horses, 703 remained after excluding duplicate records and close relatives; the X-linked LEX3 locus was excluded from population analyses. Five groups were studied: registered Kustanay (KST, n = 32), Kustanay from Qazaq Tulpary (KT, n = 36), Thoroughbred (TB, n = 31), Kustanay crossbred (CROSS, n = 9), and undocumented origin (UB, n = 595). Expected heterozygosity was high across groups (H_E_ = 0.661–0.725). Overall F_ST_ was 0.0088, and AMOVA attributed 99.13% of molecular variance to variation within individuals. The Puechmaille estimators favored *K* = 2, whereas Evanno Δ*K* peaked at *K* = 4; neither solution revealed clear separation among groups. Nei’s genetic distances and PCoA were consistent with weak differentiation and similarity between registered Kustanay and undocumented horses. Unequal group sizes limit interpretation, and these findings do not establish breed identity for individual undocumented horses. The results inform pedigree recording and conservation planning.

## Simple Summary

The Kustanay horse is a breed developed in Kazakhstan by crossing local Kazakh mares with Don, Thoroughbred, Streletskaya, and Astrakhan (Kalmyk) stallions. However, many horses considered by their owners to belong to the Kustanay breed have no official pedigree records, making their true relationship to the breed uncertain. We studied more than 700 horses from different regions of Kazakhstan, including animals with official pedigree records and horses without documented ancestry. Their genetic material was examined using 16 internationally recognized markers commonly used to compare horses and verify parentage. Group sizes were highly unbalanced: 595 horses lacked pedigree documentation, while the other groups had only 9–36 animals. The studied groups showed a high level of genetic variation and only small differences between them. Horses without pedigree documentation were especially similar to officially registered Kustanay horses. However, the absence of most founder breeds from the study does not allow this similarity to be considered evidence of Kustanay origin. Establishing the breed affiliation of individual animals requires combining genetic data with reliable pedigree information. The findings provide useful genetic information for improving pedigree records, supporting future identification studies, and helping preserve the Kustanay horse as an important genetic resource of Kazakhstan.

## 1. Introduction

Horse breeding is a strategically important sector of livestock production in the Republic of Kazakhstan, contributing to the production of horse-derived products, the advancement of pedigree and sport horse breeding, and the conservation of the nation’s equine genetic resources [1]. The Kustanay horse breed occupies a unique position among Kazakhstan’s domestic horse breeds as the first specialized riding breed developed in the country through systematic selective breeding and officially recognized in 1951 [2,3]. The Kustanay horse breed combines strong working ability, favorable athletic performance, and adaptation to the harsh continental climate of northern Kazakhstan, making it a genetic resource of considerable scientific and practical importance for the country’s horse breeding industry.

The breed was developed during the first half of the twentieth century through reproductive crossbreeding of local Kazakh steppe mares with Don, Thoroughbred, Streletskaya, and Astrakhan (Kalmyk) stallions, followed by long-term selective breeding for conformation, performance, and adaptive traits [2,4]. The Kustanay horse is a valuable indigenous genetic resource of Kazakhstan that is currently facing a serious risk of extinction. According to official statements made in November 2024, the remaining population in Kazakhstan was estimated at approximately 250–280 horses [5,6]. This small reported population size underscores the urgent need to assess genetic diversity, population structure and the potential risks associated with the loss of genetic variation.

In recent decades, the conservation of genetic diversity in local horse breeds has become increasingly important due to declining breeding populations, the widespread use of industrial crossbreeding, and the associated risk of losing unique genetic variants. In the Kustanay breed, this challenge is further complicated by the large number of animals identified as Kustanay despite lacking documented pedigree records, which hinders effective breeding management and threatens the preservation of the breed’s genetic integrity. Microsatellite genotyping has also been used to characterize the genetic diversity and population structure of other local horse breeds with documented pedigrees, such as the Lipizzan [7,8].

In accordance with the recommendations of the Food and Agriculture Organization of the United Nations (FAO) and the International Society for Animal Genetics (ISAG), monitoring of livestock genetic resources is recommended to be carried out using molecular genetic markers [9,10]. Among these, short tandem repeats (STRs) have gained the widest application; owing to their high polymorphism, codominant inheritance, and international standardization, they are used for animal identification, parentage verification, assessment of genetic diversity, and analysis of breed population structure [9,11].

Despite the widespread adoption of high-throughput SNP genotyping arrays [12], the STR marker panel used in this study—comprising 17 loci, including the 12 core markers recommended by ISAG—remains the international reference for horse identification and parentage verification [10]. In addition to individual identification, STR genotypes can be used to assess genetic diversity and population structure.

In recent years, the study of genetic resources in domestic horse breeds has advanced considerably owing to the widespread application of molecular genetic approaches. Initial investigations of the Kustanay horse breed using microsatellite markers focused on the analysis of genetic polymorphism within individual stud lines and confirmed the suitability and high discriminatory power of the standard 17-STR marker panel for genetic characterization of the breed [13]. Subsequently, a preliminary assessment of the genetic diversity of the modern Kustanay breed population was carried out on a limited sample of animals [4]. Similar studies using STR markers have been conducted for other domestic breeds, including the Kushum and Mugalzhar breeds, confirming the high effectiveness of this approach for assessing genetic diversity and population structure [14,15].

The development of genomic technologies has substantially expanded opportunities for investigating the genetic diversity and population structure of domestic horse breeds. High-density SNP genotyping arrays have been successfully applied to characterize the population structure and genetic diversity of traditional Kazakh horse populations [2,16]. In parallel, studies have been conducted on mitochondrial DNA and Y-chromosome haplotypes of the Kushum breed [17], as well as maternal diversity in domestic horse breeds [3]. Furthermore, the first high-quality de novo genome assembly of the Kazakh Zhabe horse has been reported, providing an important genomic resource for future investigations of structural variation and the functional genomics of domestic horse breeds [18].

Despite these advances, the current state of the Kustanay horse breed’s gene pool remains insufficiently characterized. Previous studies have focused primarily on individual stud lines, limited sample sizes, or other domestic horse breeds. To date, no comprehensive assessment of the contemporary Kustanay horse population has been performed using the internationally standardized ISAG panel of 17 STR markers. Furthermore, the applicability of this marker panel has not been evaluated for the molecular characterization of both registered Kustanay horses and animals classified as Kustanay despite lacking documented pedigree records. Addressing this knowledge gap is essential for improving breeding management, preserving the breed’s genetic integrity, strengthening animal identification and parentage verification systems, and providing a scientific basis for long-term conservation programs targeting Kazakhstan’s national equine genetic resources.

The aim of the present study was to comprehensively assess the genetic diversity, population structure, and degree of genetic differentiation of the Kustanay horse breed based on the analysis of 16 autosomal STR loci of a 17-locus panel that includes the 12 ISAG core markers. In addition, the study aimed to evaluate the genetic structure of horse groups differing in origin and documented breed status.

## 2. Materials and Methods

### 2.1. Sampling

The study included 765 individuals of *Equus caballus*, distributed across five groups (Table 1).

**Figure 1:**
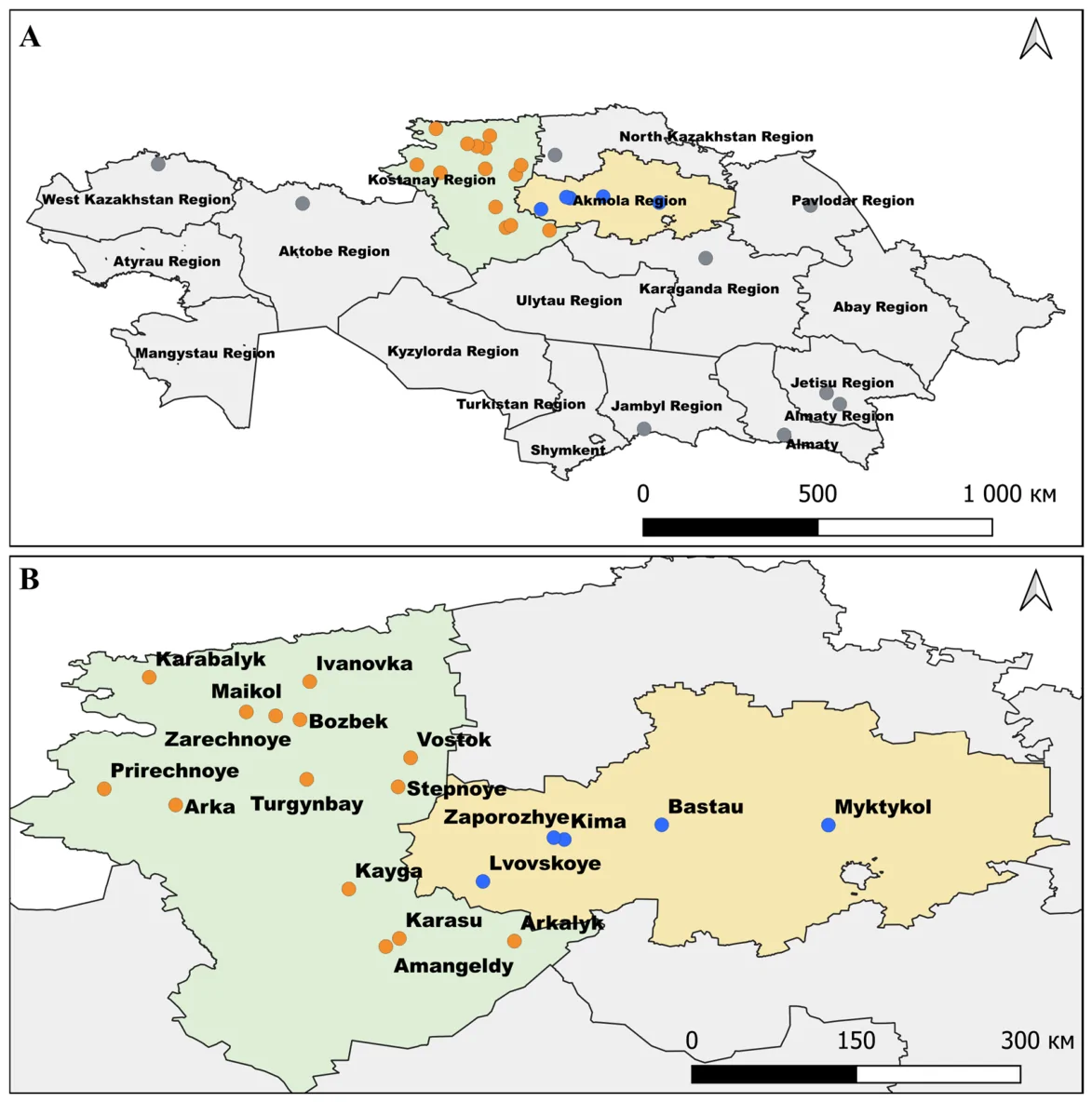
Geographic distribution of horse sampling locations in Kazakhstan. (**A**) Country-level map of Kazakhstan showing all sampling locations; Kostanay and Akmola Regions are highlighted in green and yellow, respectively. Orange and blue points indicate sampling locations in Kostanay and Akmola Regions, respectively, whereas gray points indicate sampling locations in other regions of Kazakhstan. (**B**) Detailed view of the sampling locations in Kostanay and Akmola Regions, with individual sampling localities labeled.

Sampling was carried out in 2021–2025 from adult animals of both sexes kept on stud farms, commercial farms, and private smallholdings in various regions of the Republic of Kazakhstan (Figure 1, Table S1).

Hair follicles were used as the source of genomic DNA. From each animal, 30–40 hairs with intact follicles were collected. Biological material was collected by breeding inspectors of the Republican Chamber for Pedigree Horse Breeds. The Chamber provided written consent for laboratory analysis of the collected material and for the use of the research results in scientific publications. Each sample was assigned a unique identification number and stored in an individually labeled paper envelope.

Animals were assigned to groups based on breeding certificates, pedigree records, and information provided by their owners. The UB group comprised horses identified by their owners as Kustanay horses but lacking official pedigree documentation confirming their origin. The Thoroughbred (TB) breed was included because of its historical contribution to the formation of the Kustanay breed and the availability of samples from registered horses of this breed. Other breeds that contributed to the formation of the Kustanay breed were not included in the study owing to the lack of available samples. Moreover, the study was not designed to assess the relative genetic contribution of individual founder breeds to the modern Kustanay horse population. KST comprised registered Kustanay horses from private owners, whereas KT consisted exclusively of horses from a single stud farm, “Qazaq Tulpary” LLP, which specializes in breeding the Kustanay breed. The sample size of each group was determined by the availability of animals meeting the respective inclusion criteria at the time of sampling.

The study was approved by the Local Ethics Committee of the Non-profit Limited Company “Akhmet Baitursynuly Kostanay Regional University” (Protocol No. 9, 30 September 2024). Hair follicle samples collected prior to the approval date had originally been obtained by authorized breeding inspectors of the Republican Chamber for Pedigree Horse Breeds for routine pedigree registration and genetic identification purposes, independent of the present study, and were subsequently used as archived material under the approved protocol. All sampling was performed using a non-invasive procedure and in accordance with national and institutional guidelines for the humane treatment of animals.

### 2.2. DNA Extraction

DNA extraction and genotyping were performed at the Laboratory of Molecular Genetic Research, Non-profit Limited Company “Akhmet Baitursynuly Kostanay Regional University”. Genomic DNA was extracted using the DNA-Extran-2 kit (SYNTOL LLC, Moscow, Russia) according to the manufacturer’s instructions. DNA concentration and purity were determined using a NanoDrop™ One spectrophotometer (Thermo Fisher Scientific, Waltham, MA, USA). DNA samples were stored at −20 °C until further molecular genetic analyses.

### 2.3. STR Genotyping

Genotyping was performed using the commercial “COrDIS Horse” kit (GORDIZ, Moscow, Russia), comprising a 17-locus STR panel that includes 12 core markers recommended by ISAG for genetic identification and five additional loci [10]. The panel comprised 12 core (AHT4, AHT5, ASB2, ASB17, ASB23, HMS2, HMS3, HMS6, HMS7, HTG4, HTG10, VHL20) and 5 additional (CA425, HMS1, HTG6, HTG7, LEX3) STR loci. The chromosomal location, structure, and repeat types of the 17 STR loci used in the study are presented in Table 2.

DNA amplification was performed by multiplex PCR according to the manufacturer’s instructions using a ProFlex™ PCR System thermal cycler (Applied Biosystems, Waltham, MA, USA). Each PCR run included a positive control.

PCR products were separated by capillary electrophoresis using an ABI 3500 Genetic Analyzer (Applied Biosystems, Waltham, MA, USA). Electropherograms were analyzed using GeneMapper™ Software version 6.0 (Applied Biosystems, Waltham, MA, USA).

Alleles were designated according to the international nomenclature proposed by van de Goor et al. (2010) [19]. Samples that failed to amplify or produced poor-quality electropherograms were re-amplified and re-genotyped; only profiles that passed quality control were included in the analysis. During genotype scoring, samples were identified by unique codes, and the personnel responsible for electropherogram interpretation were blinded to the animals’ study-group assignments.

The accuracy of genotyping was further assessed through the laboratory’s participation in the 2024–2025 ISAG International Horse STR DNA Typing Comparison Test (100 participating laboratories). The laboratory achieved the highest absolute genotyping accuracy rank (rank 1, 98–100%), based on the 12 core ISAG markers.

### 2.4. Data Analysis

#### 2.4.1. Data Quality Control and Compilation of the Final Dataset

All 765 samples were genotyped at all loci, including repeated genotyping (100% data completeness across loci and samples). Duplicates were identified by pairwise comparison of multilocus genotypes; pairs of animals differing at no more than two loci were rechecked against the electropherograms. The X-linked locus LEX3 was excluded from all analyses because it is hemizygous in males, which distorts estimates of heterozygosity and F. To assess the effect of its exclusion, H_O_, H_E_, F, and the STRUCTURE analysis were compared for the original 765 animals using 17 and 16 loci.

Genotyping errors due to stutter peaks and large-allele dropout, as well as null alleles, were assessed in each group using MICRO-CHECKER v2.2.3 [20] (dinucleotide repeat model, 1000 randomizations, 95% confidence interval). Null allele frequencies were estimated using the van Oosterhout, Chakraborty, and Brookfield (1 and 2) methods implemented in the program.

Close relatives were identified by sibship reconstruction in COLONY v2.0.7.2 [21] using the full-likelihood method [22,23]. Each group was analyzed separately, so that allele frequencies were estimated from the sample of that group; five runs were performed per group (seed 1234), with an allelic dropout rate of 0 and another-error rate of 0.01 for all loci. Full-sib pairs with a probability ≥ 0.95 were considered reliable. A full-sib family was defined as a connected chain of such pairs; one animal was retained from each family (the one with the lowest sample number). Half-sibs were not removed.

All subsequent analyses were performed on the final dataset (n = 703, 16 autosomal loci) unless stated otherwise. Deviations from Hardy–Weinberg equilibrium (HWE) and linkage disequilibrium (LD) were tested in each group in R v4.5.2–4.6.1 [24] using the genepop package v1.2.17 [25]. HWE was tested for each locus using an exact test with the *p* value estimated by a Markov chain method [26]; genotypic LD was tested for all 120 pairs of loci in each group. Chain parameters were 10,000 dememorization steps and 100 batches of 5000 iterations (random seed 67144630). *p* values were Bonferroni-corrected within each group (16 tests for HWE, 120 for LD).

#### 2.4.2. Assessment of Diversity and Differentiation

Genetic diversity was assessed using GenAlEx version 6.51 software [27]. For each study group, the following parameters were calculated: allele frequencies (Table S2), number of alleles per locus (Na), effective number of alleles (Ne), Shannon’s information index (I), observed (H_O_) and expected (H_E_) heterozygosity, unbiased expected heterozygosity (uH_E_), the fixation index (F), and Nei’s genetic distances [28].

Means and SE for each study group were calculated across the 16 STR loci. To account for differences in sample size, allelic richness (Ar) and private allelic richness (PAr) were estimated by rarefaction in ADZE v1.0 [29], standardized to the smallest sample (g = 18 gene copies, corresponding to the CROSS group, n = 9).

Differentiation was assessed using the multilocus F_ST_ statistic of Weir and Cockerham [30]. Overall F_ST_, F_IS_, and F_IT_, as well as their per-locus values, were obtained by analysis of molecular variance (AMOVA) in GenAlEx (allelic distance); pairwise F_ST_ were obtained in the same way. Significance was tested with 999 permutations, with Bonferroni correction for 16 loci and 10 pairs of groups. The same estimates were calculated in R using the hierfstat package v0.5-11 [31]; 95% confidence intervals (CI) for overall and pairwise F_ST_ were obtained by bootstrapping over loci (1000 replicates). The number of migrants per generation was calculated from multilocus F_ST_ under Wright’s island model [32]: Nm = (1/F_ST_ − 1)/4; the CI for Nm was obtained by applying the same transformation to the CI limits of F_ST_.

#### 2.4.3. Analysis of Genetic Structure

Cluster analysis was performed using STRUCTURE version 2.3.4, based on a Bayesian algorithm and the Markov chain Monte Carlo (MCMC) method, under the admixture model with independent allele frequencies, without prior group information [33]. The number of genetic clusters (*K*) was evaluated from 1 to 10, with 10 independent runs conducted for each *K* value. Each run included 250,000 burn-in iterations, followed by 1,000,000 MCMC iterations.

Principal coordinates analysis (PCoA) was performed based on a matrix of Nei’s genetic distances using GenAlEx version 6.51 software [27].

The number of clusters was estimated in StructureSelector [34] using two approaches: the Δ*K* method [35] and four Puechmaille estimators [36] (MedMedK, MedMeanK, MaxMedK, MaxMeanK). Replicate runs were aligned and modes were identified in CLUMPAK [37] (CLUMPP, LargeKGreedy algorithm, 2000 repeats; MCL threshold 0.1). For the comparison of 17 and 16 loci, runs on all 765 animals were used.

As a non-Bayesian complement to STRUCTURE, discriminant analysis of principal components (DAPC) [38] was performed in R with the adegenet package v2.1.11 [39]. First, the number of clusters was estimated without group information: k-means clustering on the principal components of allele frequencies for *K* = 1–10 (100 random starts), compared by the Bayesian information criterion (BIC) using the “diffNgroup” selection criterion. For *K* = 2–5, DAPC was performed on the inferred clusters and plots of individual membership probabilities were produced. DAPC was then performed using the five predefined groups. In both cases, the number of principal components was selected by cross-validation (xvalDapc; 90% training set, 30 repetitions, lowest mean squared error). Assignment accuracy was evaluated by leave-one-out cross-validation of linear discriminant analysis on the retained components (MASS package v7.3-66 [40], with either equal prior probabilities for all groups or priors proportional to group sizes).

An individual-level assignment test and PCoA were also performed in GenAlEx version 6.51. The assignment test was based on the log-likelihood of the multilocus genotype in each group; the tested individual was excluded from the allele frequency calculation (leave-one-out), and zero frequencies were replaced with 0.01. The individual-level PCoA was based on the genetic distance matrix for codominant markers, using the standardized covariance matrix.

To assess the effect of unequal sample sizes, 10 balanced datasets were created: 36 animals (the size of the largest documented group, KT) were randomly drawn without replacement from the UB group and combined with all animals of the other groups, giving 144 animals per dataset. For each dataset, STRUCTURE (*K* = 1–6), DAPC, and overall F_ST_ were calculated with the same settings as for the full dataset.

## 3. Results

### 3.1. Data Quality Control

Data quality control (MICRO-CHECKER and comparison of multilocus genotypes) identified five HTG4 genotypes carrying an allele inconsistent with a dinucleotide repeat, and four pairs of nearly identical genotypes. After re-examination of the electropherograms at these loci, the HTG4 genotypes were corrected, and the four pairs proved to be repeated samples of the same animals; the duplicate records were removed. The further analysis included 761 animals (CROSS 9, KST 32, KT 39, TB 31, UB 650).

On the corrected data, MICRO-CHECKER detected no evidence of stutter peaks or large-allele dropout at any of the 16 loci in any group. A null-allele signal was detected only at HMS3 in the UB group (0.0230–0.0305; Table S3); HMS3 was therefore retained in the analysis.

COLONY detected no duplicated genotypes in any group. The final log-likelihoods of the five runs were identical in CROSS (−284.77) and differed by less than 3.2 units in KT, TB, and KST. In UB the difference reached 73 units (from −21,863.3 to −21,936.4), but the number of full-sib pairs in the best configuration was similar across runs (173–182). Full-sib pairs with a probability ≥ 0.95 were found only in KT (3 pairs, three families of two animals) and UB (59 pairs, 95 animals in 40 families of 2–5 animals). In the other groups, the highest full-sib pair probability was 0.826 (KST), 0.276 (CROSS), and 0.261 (TB). Half-sib pairs with a probability ≥ 0.95 were found only in UB (8 pairs). After removal of 3 animals from KT and 55 from UB, 703 animals remained in the analysis (UB 595, KT 36, KST 32, TB 31, CROSS 9; Table S4).

After Bonferroni correction, no locus deviated from HWE in any group (Table S5). Before correction, *p* < 0.05 was observed in only three of 80 locus × group combinations (HMS3 and ASB2 in UB, HTG7 in KST). Significant LD after correction was found only in UB (17 of 120 locus pairs) and in none of the other groups (Table S6). Because LD in UB involved many different locus pairs and was not observed in the other groups, it more likely reflects the heterogeneity of the UB group than physical linkage between loci.

In the original 765 animals, excluding LEX3 changed H_O_ and H_E_ little but removed the heterozygote deficit: F in TB decreased from 0.029 to 0.002, and in UB from 0.014 to −0.010 (Table S7). At the LEX3 locus itself, F ranged from 0.16 (CROSS) to 0.46 (TB).

### 3.2. Allelic Diversity and Allele Frequencies

Analysis of allele frequencies across the 16 STR loci revealed a high level of polymorphism in all studied groups (Table S8). A total of 155 distinct alleles were detected across the analyzed loci, and the complete allele frequency distribution is provided in Table S2. The number of alleles detected per locus ranged from 2 (HTG7 in the CROSS group) to 16 (ASB17 in the UB group). The most polymorphic loci were ASB17, HTG10, ASB2, ASB23, and CA425, whereas HTG7 exhibited the lowest level of polymorphism.

The greatest allelic diversity was observed in the UB group, which displayed the broadest allelic spectrum among all studied groups. In contrast, the groups with documented breed affiliation (KST, KT, and TB) exhibited fewer alleles. Most rare alleles occurred at frequencies below 5%, whereas a limited number of common alleles accounted for the majority of the allelic spectrum at each locus.

Despite differences in the number of alleles, the distribution of the most frequent alleles was similar across all studied groups (Table S2).

At the AHT4 locus, allele 157 was the most frequent allele in four of the five groups, whereas allele 149 predominated only in the CROSS group. Similarly, alleles 222, 238, and 148 were the most frequent alleles at the HMS2, CA425, and HMS3 loci, respectively.

The UB group displayed a greater diversity of rare alleles that were either absent or present at low frequency in the other groups. This feature indicates a broader allelic spectrum within the studied sample and provided the basis for its further analysis using genetic diversity indices and population structure analyses.

### 3.3. Genetic Diversity Indices

The average values of genetic diversity indices, calculated across the 16 STR loci, are presented in Table 3.

The highest mean number of observed alleles (Na) was recorded in the UB group (9.688 ± 0.669), whereas the remaining groups exhibited values ranging from 4.250 ± 0.296 (CROSS) to 6.438 ± 0.387 (KT).

After rarefaction to 18 gene copies, Ar ranged from 4.25 (CROSS) to 4.97 (UB) and differed little among the groups (KST 4.67, TB 4.54, KT 4.95). Therefore, the high Na in UB mainly reflects its sample size: after rarefaction, Ar in UB was almost equal to that in KT. Private allelic richness was low in all groups (0.12–0.41 alleles per locus): it was highest in KT (0.41) and UB (0.35) and lowest in TB (0.12). The effective number of alleles ranged from 3.260 ± 0.263 (CROSS) to 3.974 ± 0.314 (UB), and Shannon’s information index from 1.244 ± 0.077 (CROSS) to 1.530 ± 0.070 (UB).

Heterozygosity was high in all groups: H_O_ ranged from 0.696 ± 0.030 (TB) to 0.736 ± 0.024 (KT), H_E_ from 0.661 ± 0.028 (CROSS) to 0.725 ± 0.021 (UB), and uH_E_ from 0.700 ± 0.030 (CROSS) to 0.725 ± 0.021 (UB). The fixation index was close to zero or negative in all groups, ranging from −0.099 (CROSS) to 0.002 (TB); it was −0.025 in KST, −0.045 in KT, and −0.011 in UB. No pronounced heterozygote deficiency was observed in any group.

All groups had high heterozygosity and similar allelic richness after correction for sample size.

### 3.4. Genetic Differentiation Among the Studied Groups

F-statistics (F_IS_, F_IT_ and F_ST_) were estimated by AMOVA for each locus and across all loci and are presented in Table 4.

Locus-specific F_ST_ ranged from −0.001 (HMS3) to 0.043 (HTG4). Differentiation was significant at eight loci (*p* < 0.05) and remained significant after Bonferroni correction at AHT4 and HTG4. Across all loci, differentiation was low but significant (F_ST_ = 0.0088, 95% CI 0.0051–0.0142; *p* = 0.001), and there was no heterozygote deficiency within groups (F_IS_ = −0.0095; *p* = 0.921) (Table 5).

The number of migrants per generation, calculated from the overall F_ST_ (Table 5) and its 95% CI (Table 4), was Nm = 28.1 (95% CI 17.4–48.6).

According to the AMOVA results, 0.87% of the total variation was attributable to differences among groups and 99.13% to variation within individuals; the component among individuals within groups was zero (Table 5). The Weir and Cockerham estimates obtained in hierfstat agreed with the AMOVA estimates.

Pairwise F_ST_ ranged from 0.0017 (KST–UB) to 0.0463 (CROSS–KT) (Table 6). After Bonferroni correction, differentiation was significant for the pairs KT–TB, CROSS–KT, TB–UB, and CROSS–UB; the 95% CIs included zero for the pairs KST–UB and CROSS–TB. Because the CROSS group is small (n = 9), F_ST_ values for pairs involving this group should be interpreted with caution.

In the 10 balanced subsamples, the overall F_ST_ ranged from 0.0120 to 0.0157 (mean 0.0136), and none of the 95% CIs included zero (lower limits 0.0064–0.0095) (Table S9; composition of the subsamples in Table S10). These values were slightly higher than in the full dataset, in which the estimate is dominated by the large UB group, but remained low. Thus, differentiation among the studied groups was low but statistically significant, and this conclusion does not depend on unequal sample sizes.

### 3.5. Genetic Structure of the Studied Groups

**Figure 2:**
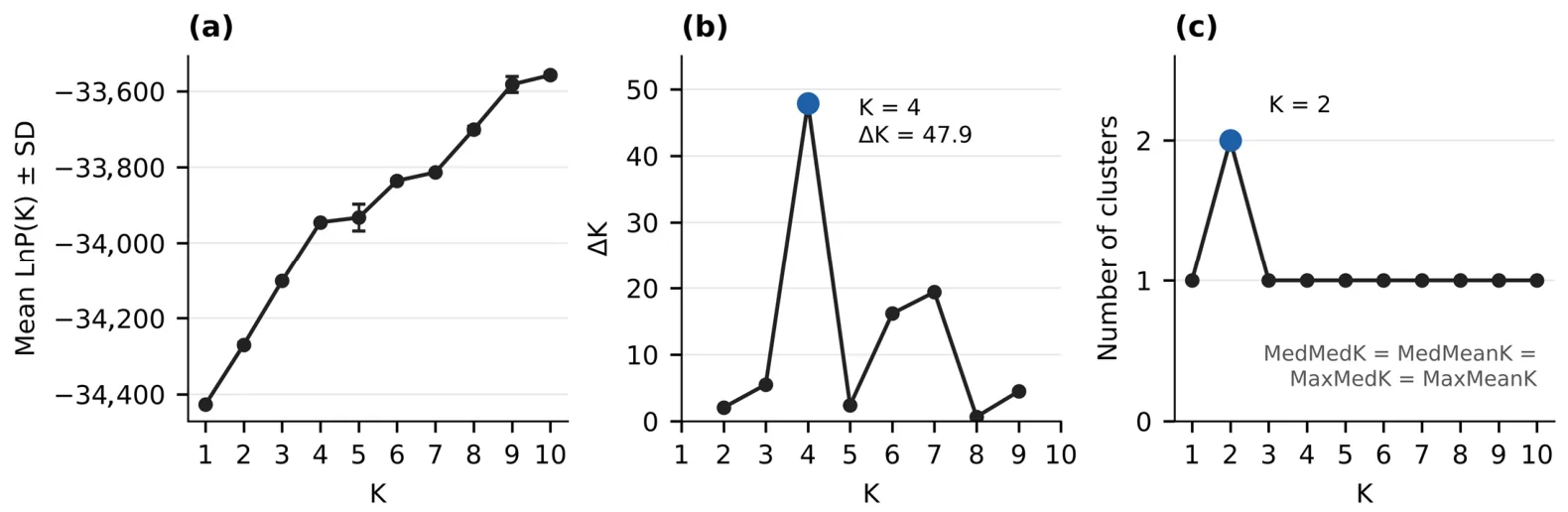
Selection of the number of genetic clusters (*K*) for the final dataset (n = 703): (**a**) mean Ln*P*(*K*) ± SD over 10 runs; (**b**) Δ*K* [35]; (**c**) Puechmaille estimators [36]. MedMeanK and MaxMeanK are the median and the maximum across runs of the number of clusters in which at least one predefined group has a mean membership coefficient > 0.5; MedMedK and MaxMedK are the same quantities calculated using the median membership coefficient of each group.

The mean log-likelihood, Ln*P*(*K*), increased monotonically from *K* = 1 (−34,427.2 ± 0.05) to *K* = 10 (−33,557.4 ± 6.7) without reaching a plateau (Table S11). All four Puechmaille estimators indicated *K* = 2, whereas Δ*K* was highest at *K* = 4 (Δ*K* = 47.9), with smaller peaks at *K* = 7 (19.4) and *K* = 6 (16.2) (Figure 2). The Δ*K* peak at *K* = 4 coincided with a sharp increase in variation among runs at *K* = 5 (SD of Ln*P*(*K*) = 35.2 versus 2.1–5.3 at *K* = 2–4). CLUMPAK identified a single mode across all 10 runs at *K* = 1–4, 6, and 9, and two to three modes at *K* = 5, 7, 8, and 10.

**Figure 3:**
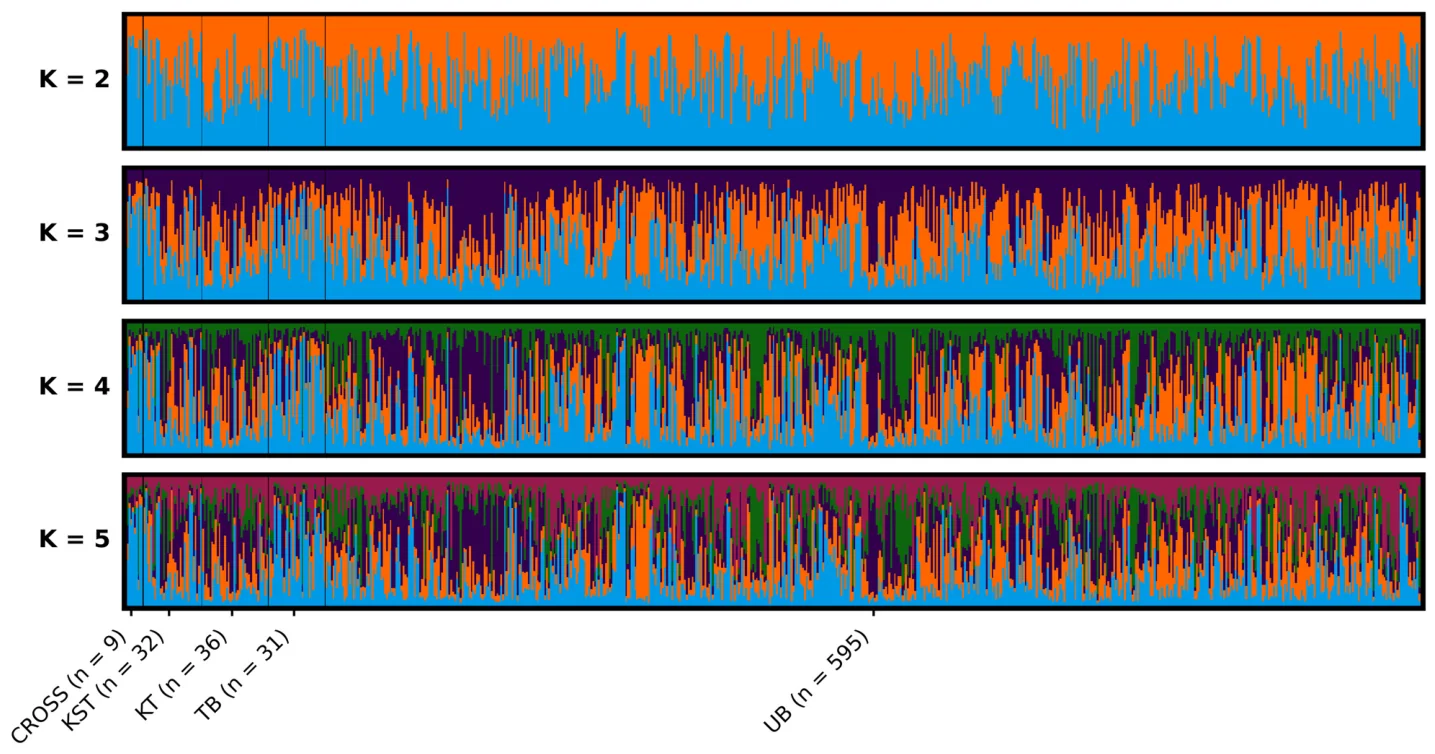
STRUCTURE results at *K* = 2–5 (n = 703). Each bar represents one animal; colors indicate membership coefficients for the clusters.

Individual membership coefficients were highly admixed at all values of K (Figure 3). At *K* = 2, the mean proportion of the first cluster was 0.72 in CROSS, 0.67 in TB, 0.59 in KST, 0.51 in UB, and 0.43 in KT; the second cluster exceeded the 0.5 threshold only in KT (0.57), and only 14.9% of the animals had a membership coefficient ≥ 0.8 to one of the clusters. At *K* = 3–5, the mean membership of UB was close to 1/K for each cluster, and no group formed a separate cluster.

The Puechmaille estimators indicated *K* = 2 in the original 17-locus dataset and in both 16-locus datasets, and the mean membership of the groups remained practically unchanged. With LEX3, Δ*K* was also highest at *K* = 2 (Δ*K* = 94.0); after exclusion of this locus, this peak disappeared (Δ*K* = 0.9–2.0), and the highest Δ*K* shifted to *K* = 4. In none of the datasets did the identified clusters correspond to the predefined groups (Table S7).

In the 10 balanced subsamples (n = 144), the Puechmaille estimators indicated *K* = 2 in all cases, and the mean membership of the groups at *K* = 2 was close to that in the full dataset (Table S12). In contrast, the maximum Δ*K* differed among subsamples (*K* = 2 in two, *K* = 3 in five, *K* = 4 in three), and in five subsamples the mean LnP at *K* = 2 was lower than at *K* = 1. Thus, equalizing group sizes did not reveal additional structure.

BIC decreased gradually with increasing K, from 1235.9 (*K* = 1) to a minimum of 1194.9 (*K* = 9), with a difference of less than 6 units after *K* = 5 and without a clear inflection point (Figure S1). The diffNgroup criterion indicated *K* = 2, but the two clusters found did not correspond to the predefined groups: KST animals were split equally (16 and 16), and UB animals in similar proportions (266 and 329) (Table S13). At *K* = 3–5, individuals of all groups showed mixed membership (Figure S2).

In the DAPC with predefined groups (80 principal components, 98% of variance), leave-one-out assignment with equal prior probabilities correctly assigned 33.3% of CROSS, 40.6% of KST, 50.0% of KT, 45.2% of TB, and 52.8% of UB (Table S13). With prior probabilities proportional to group sizes, 92.8% of UB animals were correctly assigned, but most animals of the small groups were assigned to UB (correct assignment 3.1–33.3%). In the subsamples, DAPC also did not yield a stable number of clusters: diffNgroup indicated *K* = 2 in two subsamples, *K* = 3 in three, *K* = 4 in three, and *K* = 5 in two, and the minimum BIC was at *K* = 3 in eight subsamples and at *K* = 2 in two. The mean proportion of correct assignment with equal prior probabilities was 45.5% for CROSS (range 33.3–55.6%), 30.0% for KST (21.9–40.6%), 57.5% for KT (50.0–66.7%), 37.4% for TB (22.6–48.4%), and 32.2% for UB (19.4–44.4%) (Table S9).

The assignment test with exclusion of the tested individual correctly assigned 240 of 703 animals (34.1%) to their own group: 55.6% of CROSS, 40.6% of KST, 50.0% of KT, 38.7% of TB, and 32.3% of UB (Table 7). UB animals were distributed among all groups: 136 to KST, 109 to KT, and 143 to TB. The proportions of correct assignment for KST, KT, and TB were close to the DAPC results with equal prior probabilities.

**Figure 4:**
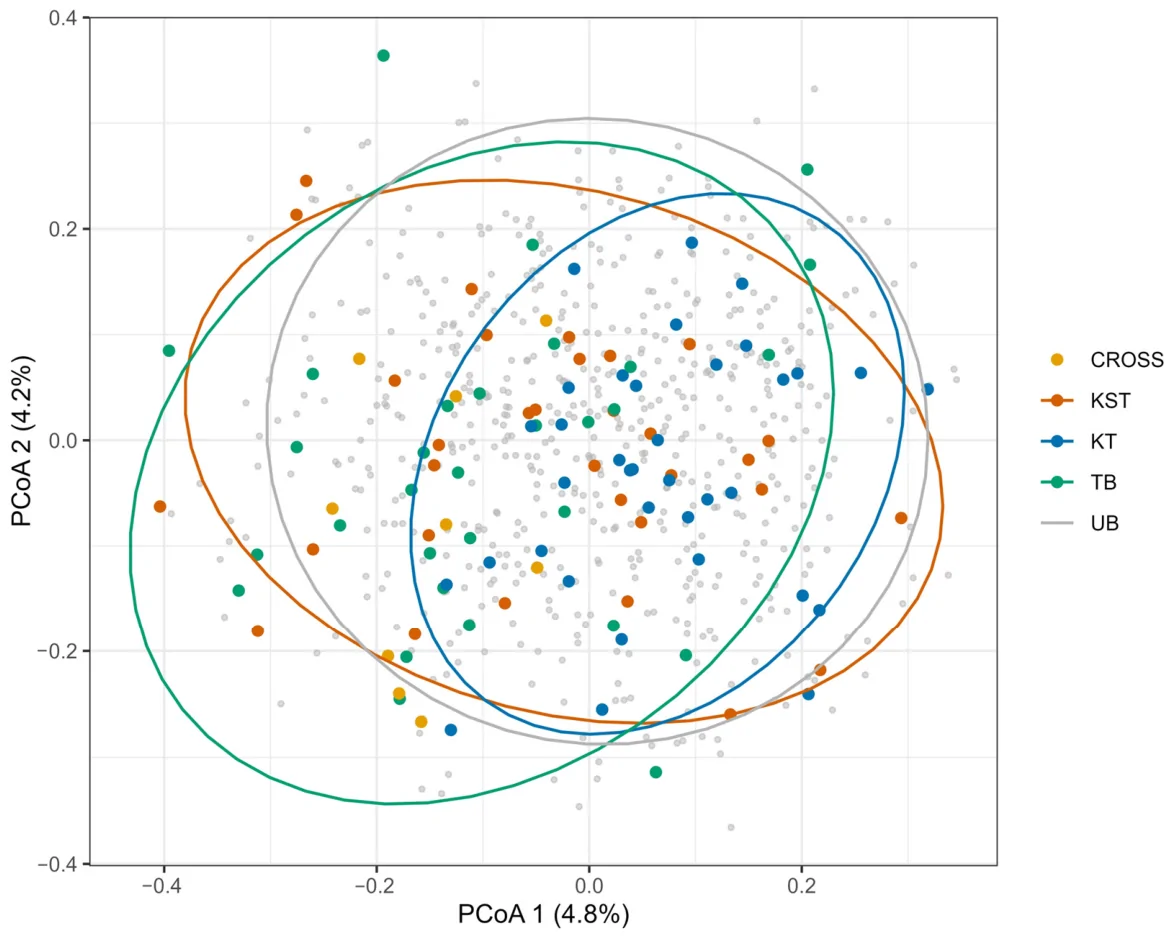
Principal coordinates analysis of individuals (n = 703). UB animals are shown in gray; 95% ellipses are shown for all groups except CROSS (n = 9).

The first two axes of the individual-level PCoA explained 4.8% and 4.2% of the variation (three axes, 12.9%). The 95% ellipses of the groups almost completely overlapped; only TB was slightly shifted toward negative values of the first axis, remaining within the overall cloud of points (Figure 4).

Pairwise Nei’s genetic distance analysis likewise demonstrated a high degree of genetic similarity among the studied groups (Table 8).

All pairwise genetic distances were low, ranging from 0.024 to 0.201, indicating only weak genetic differentiation among the studied groups.

The smallest genetic distance was observed between the KST and UB groups (0.024), followed by the KT and UB groups (0.040) and the TB and UB groups (0.043), indicating a high degree of genetic similarity among these groups. Relatively small genetic distances were also recorded between the KST and KT groups (0.061) and between the KST and TB groups (0.057). The largest genetic distance was observed between the CROSS and KT groups (0.201). Likewise, the genetic distances between the CROSS group and the other groups (0.093–0.201) were higher than those observed for most other group pairs. When interpreting the results, the small sample size of the CROSS group (n = 9), which may be insufficient for estimating genetic distances, must be taken into account; therefore, the observed genetic remoteness of the CROSS group should be interpreted with caution and not as evidence of unambiguous genetic differentiation. The genetic similarity between KST and UB indicates some shared genetic characteristics between the two groups; however, the genetic heterogeneity and potentially different origin of the UB horses must be taken into account.

**Figure 5:**
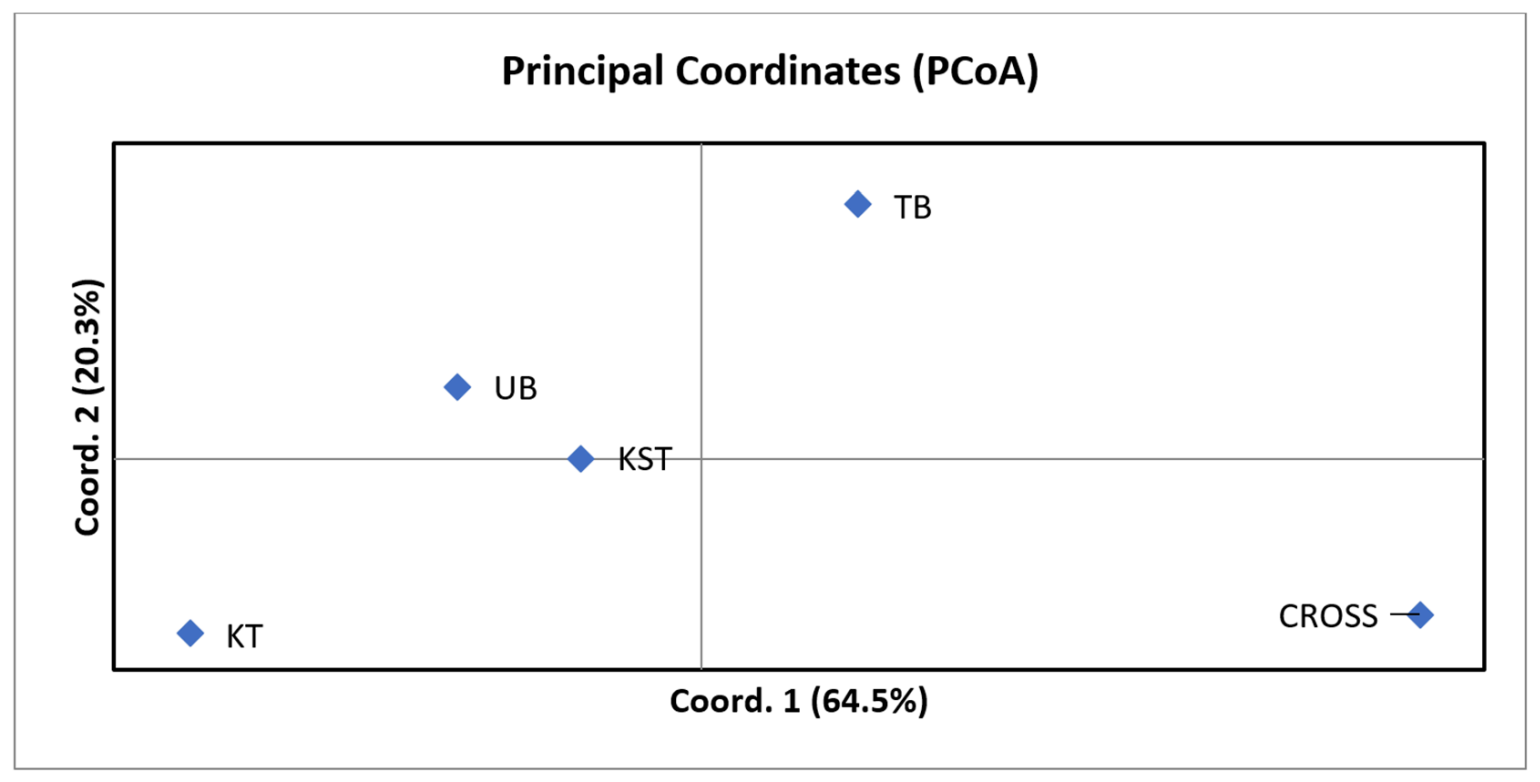
Principal coordinates analysis (PCoA) based on the matrix of Nei’s genetic distances among the five studied horse groups.

The results of the principal coordinates analysis (PCoA) were consistent with those obtained from Nei’s genetic distance and STRUCTURE analyses (Figure 5).

The studied groups did not form clearly distinct clusters, and their distribution in the principal coordinate space was characterized by substantial overlap. The KST and UB groups were located in close proximity, consistent with the minimal Nei’s genetic distance observed between them. Along the first coordinate (64.5% of the variation), CROSS and KT occupied the extreme positions, whereas KST and UB were located centrally and close to each other; TB occupied an intermediate position on the first coordinate and was displaced along the second coordinate (20.3%). However, the spatial separation among groups remained limited and was not accompanied by the formation of distinct clusters. It should be taken into account that the CROSS group is represented by a small number of individuals (n = 9), which limits the reliability of the interpretation of the results. Therefore, the remoteness of the CROSS group revealed by the PCoA cannot unambiguously indicate its genetic distinctness.

Overall, STRUCTURE, DAPC, the assignment test, individual-level PCoA, and Nei’s genetic distances consistently show low differentiation: none of the methods identified the studied groups as separate genetic clusters, which is consistent with the AMOVA results.

## 4. Discussion

### 4.1. Genetic Diversity of the Kustanay Horse Breed

The high level of allelic polymorphism observed across all studied horse groups confirms the suitability of 17-locus STR panel including the 12 ISAG core markers for assessing genetic diversity in the Kustanay horse breed. Comparable applications of microsatellite markers have been reported for other horse breeds, where they have proved informative for evaluating genetic diversity and population structure [41,42,43]. The present results are also consistent with previous studies of the Kustanay breed [4,13], while substantially expanding the available information by including 765 genotyped animals (703 after quality control and removal of close relatives), comprising both registered horses and animals of undocumented origin.

The UB group had the highest number of alleles, including all alleles found in the study. However, after rarefaction to 18 gene copies, allelic richness in UB was practically equal to that in KT. Therefore, the larger number of alleles in UB is explained mainly by sample size rather than by a broader allelic spectrum. Similar patterns of substantial genetic diversity have also been reported in Kazakh and other domestic horse populations using high-density SNP genotyping [2,16] and mitochondrial DNA analyses [3]. Although these marker systems characterize different components of genetic variation, together they indicate that considerable genetic diversity remains within domestic horse populations.

The obtained heterozygosity values were comparable to the results of previous studies of the Kustanay breed using the same 17-marker panel [4,13] and fall within the range typical of other horse breeds, including the Kushum, Mugalzhar, Akhal-Teke, and Thoroughbred breeds [14,15,43,44].

Fixation indices were close to zero or negative in all groups, and the overall F_IS_ from AMOVA did not differ from zero. The slight heterozygote deficit in TB and UB in the original analysis was associated with the X-linked locus LEX3: after its exclusion, F decreased in the same 765 animals. Male horses are hemizygous at this locus, and recording their genotypes as apparent homozygotes can artificially inflate estimates of heterozygote deficiency. Overall, the fixation indices did not indicate a pronounced heterozygote deficit. Similar effects have been reported in Arabian horses genotyped with the same microsatellite panel [45]. The absence of deviations from HWE after correction for multiple comparisons and the low frequency of null alleles (only HMS3 in UB) indicate no heterozygote deficit attributable to inbreeding in the final dataset (from which close relatives had been removed) and the reliability of genotyping.

### 4.2. Genetic Differentiation and Population Structure

The multilocus F_ST_ indicates weak differentiation among the groups according to Wright’s classification [46]; in the balanced subsamples, it was slightly higher but remained low. The Nm estimate, calculated from F_ST_ under the island model, is consistent with the observed low genetic differentiation but should not be interpreted as independent evidence of contemporary gene flow or panmixia. This estimate depends on the assumptions of the underlying model. The observed differentiation may reflect both historical gene exchange and the recent common origin of the groups. In addition, differences in sample sizes and the possible heterogeneity of the UB group must be taken into account. Therefore, the available data do not allow definitive conclusions about the intensity of current gene flow among the studied groups.

AMOVA supported the same general pattern. In the complete dataset, only 0.87% of the total variation was attributable to differences among groups, whereas 99.13% occurred within individuals.

These findings are consistent with previous studies of domestic horses. Orazymbetova et al. [14], using STR markers, and Cosgrove et al. [47], using SNP genotyping and AMOVA, reported that 86% of the genetic variation was distributed within individuals in a comparative analysis involving Arabian horses and other breeds. Studies of Kazakh horses based on SNP data [2] and mitochondrial DNA [3] have similarly demonstrated substantial genetic diversity combined with relatively weak differentiation among populations. Such patterns are compatible with the complex breeding history of Kazakh horses and the historical exchange of genetic material among breeding populations. When interpreting the results of STR genotyping, the possibility of homoplasy must be taken into account. Therefore, the low F_ST_ values obtained at microsatellite loci cannot serve as definitive evidence of genetic homogeneity of the studied groups. More accurate assessment of genetic structure requires further studies using high-density SNP arrays.

The low level of differentiation observed in the present study may also reflect the formation history of the Kustanay breed. The breed originated through prolonged crossbreeding of local Kazakh mares with Don, Thoroughbred, Streletskaya, and Astrakhan (Kalmyk) stallions, followed by long-term selection within a relatively interconnected breeding population [2,4]. The exchange of breeding stallions among farms and the repeated use of influential sire lines may also have contributed to the maintenance of a shared genetic background and limited the development of strongly differentiated subpopulations.

The two approaches to choosing *K* gave different results (*K* = 2 by the Puechmaille estimators and *K* = 4 by Δ*K*), but neither solution corresponded to the predefined groups. With weak differentiation and unequal sample sizes, Δ*K* tends to produce spurious peaks and cannot evaluate *K* = 1, whereas the Puechmaille estimators are more robust to unequal sample sizes [36,48]. In our data, the solution obtained with the Puechmaille estimators was characterized by only moderate membership of animals in the identified clusters, and Ln*P*(*K*) increased without reaching a plateau. In half of the balanced subsamples, LnP at *K* = 2 was lower than at *K* = 1. Together with the absence of a clear BIC minimum in DAPC and the low assignment accuracy in GenAlEx, this shows that the studied groups do not form separate genetic clusters. A similar combination of STRUCTURE, PCoA, and phylogenetic approaches also revealed weak subdivision among closely related native horse breeds from Xinjiang [49].

Nei’s genetic distances were consistent with the close genetic relationships among the studied groups. The smallest distance was observed between KST and UB; KT and TB were similarly close to UB. In the PCoA, KST and UB were located close together in the central part of the plot, whereas CROSS and KT occupied the extreme positions along the first coordinate (Figure 5). The position of CROSS should be interpreted with caution because of the small sample (n = 9). These differences were small and did not lead to the formation of clearly separated clusters.

The small genetic distance between the KST and UB groups indicates their genetic similarity at the group level. This result does not confirm that individual animals from the UB group belong to the Kustanay breed. The overall accuracy of individual assignment was 34.1%, which indicates the limited ability of the markers used to distinguish the studied groups. Despite leave-one-out cross-validation, the results obtained are insufficient to reliably confirm the breed status of individual animals without documented origin. Therefore, molecular genetic data should be interpreted together with reliable pedigree records. Assessing the possibility of individual breed assignment requires further studies using registered Kustanay horses as a reference group.

Overall, the complementary results of F_ST_, AMOVA, STRUCTURE, DAPC, the assignment test, Nei’s genetic distances, and PCoA consistently indicate substantial genetic diversity together with weak differentiation among the studied groups. The agreement among these independent analytical approaches strengthens the conclusion that the studied groups share a largely common genetic background while retaining some differences related to their breeding history. The consistency in the distribution of the predominant alleles suggests the presence of a shared genetic background among the studied groups.

### 4.3. Study Limitations and Future Directions

Several limitations should be considered when interpreting the present results. First, the study groups differed substantially in sample size, particularly because of the large UB group (n = 595 after removal of close relatives).

To account for this, diversity was compared using rarefied allelic richness, and STRUCTURE, DAPC, and F_ST_ were repeated on 10 balanced subsamples; the conclusions did not change. Second, the CROSS group is represented by only nine animals. This reduces the power of tests for null alleles and HWE, and estimates of relatedness and allele frequencies in this group are based on a very small number of individuals; therefore, the results for CROSS should be interpreted with caution.

Despite these limitations, the large overall dataset and the consistency of results obtained using several complementary approaches provide a robust population-level characterization of the contemporary Kustanay horse gene pool. Future studies should increase the number of documented Kustanay animals, and complement STR analysis with high-density SNP genotyping. Integration of molecular data with detailed pedigree information and mitochondrial DNA may further improve estimates of effective population size, ancestry, and inbreeding. Such combined approaches have been successfully applied to the Sardinian Anglo-Arab horse [50] and the Pura Raza Español breed [51] and could provide a more detailed framework for the long-term genetic management and conservation of the Kustanay horse.

## 5. Conclusions

The present study represents the most comprehensive analysis to date of the genetic diversity and population structure of the Kustanay horse breed, based on the genotyping of 765 animals using a 17-locus STR panel including the 12 core markers recommended by ISAG (703 animals and 16 autosomal loci after quality control). A high level of genetic diversity, comparable to values reported for other horse breeds, was found in all studied groups. However, heterozygosity values alone do not allow conclusions about the preservation of the entire genetic potential of the Kustanay breed.

A comprehensive analysis incorporating genetic diversity indices, F_ST_, AMOVA, Nei’s genetic distances, Bayesian clustering (STRUCTURE), and principal coordinates analysis (PCoA) revealed only weak genetic differentiation and indicated a largely shared genetic background among the studied groups. The high Nm value is consistent with low genetic differentiation but does not allow unambiguous conclusions about the intensity of historical or contemporary gene flow among the studied groups. The greatest genetic similarity was observed between the registered Kustanay horse (KST) and UB groups. However, because most of the founder breeds were not included in the study, it cannot be excluded that this similarity reflects a broader genetic background of Kazakh horses. Thus, the results characterize genetic relationships at the group level but do not confirm that individual UB animals belong to the Kustanay breed. The limited accuracy of individual assignment underlines the need to combine molecular genetic data with reliable pedigree information.

The results support the applicability of the ISAG STR marker panel for assessing genetic diversity in the Kustanay horse breed, in addition to its established use for individual identification and parentage verification.

The data obtained substantially expand the existing knowledge of the genetic structure of the Kustanay horse breed and provide a basis for further studies. Their practical application for clarifying the origin and pedigree registration of individual animals requires further studies that include founder breeds and other genetically close groups, together with a combined analysis of molecular genetic data and reliable pedigree records.

## Figures and Tables

**Table 1:** Description of the studied horse groups (n, number of animals; before quality control).

Group Code	n	Males	Females	Sex Not Specified	Group/Breed
CROSS	9	3	6	0	Kustanay crossbred horses
KST	32	7	23	2	Registered Kustanay horses
KT	39	15	24	0	Kustanay horses from “Qazaq Tulpary” LLP
TB	31	16	15	0	Registered Thoroughbred horses
UB	654	236	374	44	Horses classified as Kustanay by owners but lacking pedigree documentation
Total	765	277	442	46	

**Table 2:** Description of the microsatellite markers.

Locus	Chromosomal Location	Repeat Type	Repeat Unit Structure
AHT4	24q14	compound	(AC)_n_AT(AC)_n_
AHT5	8	simple	(GT)_n_
ASB2	15q21.3–q23	simple	(GT)_n_
ASB17	2p14–p15	simple	(AC)_n_
ASB23	3q22.1–q22.3	simple and compound	(TG)_n_ and (TG)_n_TT(TG)_4_
CA425	28q18	simple	(GT)_n_
HMS1	15	simple	(TG)_n_
HMS2	10	compound	(CA)_n_(TC)_2_
HMS3	9	compound	(TG)_2_(CA)_2_TC(CA)_n_ and (TG)_2_(CA)_2_TC(CA)_n_GA(CA)_5_
HMS6	4	simple	(GT)_n_
HMS7	1q25	compound	(AC)_2_(CA)_n_
HTG4	9	compound	(TG)_n_AT(AG)_5_AAG(GA)_5_ACAG(AGGG)_3_
HTG6	15q26–q27	simple	(TG)_n_
HTG7	4	simple	(GT)_n_
HTG10	21	simple and compound	(TG)_n_ and TATC(TG)_n_
LEX3	Xq	simple	(TG)_n_
VHL20	30	simple	(TG)_n_

**Table 3:** Mean (±SE) values of genetic diversity parameters across 16 STR loci in the five studied horse groups.

Group (n)	Na	Ar	PAr	Ne	I	H_O_	H_E_	uH_E_	F
CROSS (9)	4.250 ± 0.296	4.250 ± 0.296	0.158 ± 0.049	3.260 ± 0.263	1.244 ± 0.077	0.729 ± 0.042	0.661 ± 0.028	0.700 ± 0.030	−0.099 ± 0.040
KST (32)	6.063 ± 0.335	4.673 ± 0.239	0.213 ± 0.064	3.558 ± 0.233	1.402 ± 0.064	0.715 ± 0.025	0.700 ± 0.020	0.711 ± 0.020	−0.025 ± 0.029
KT (36)	6.438 ± 0.387	4.948 ± 0.216	0.413 ± 0.091	3.575 ± 0.219	1.452 ± 0.056	0.736 ± 0.024	0.705 ± 0.018	0.715 ± 0.018	−0.045 ± 0.026
TB (31)	5.688 ± 0.405	4.537 ± 0.265	0.124 ± 0.037	3.567 ± 0.259	1.376 ± 0.073	0.696 ± 0.030	0.695 ± 0.024	0.706 ± 0.024	0.002 ± 0.022
UB (595)	9.688 ± 0.669	4.969 ± 0.253	0.346 ± 0.066	3.974 ± 0.314	1.530 ± 0.070	0.732 ± 0.021	0.725 ± 0.021	0.725 ± 0.021	−0.011 ± 0.009

n, number of animals; Na, number of different alleles; Ar, allelic richness; PAr, private allelic richness; Ne, number of effective alleles; I, Shannon’s information index; H_O_, observed heterozygosity; H_E_, expected heterozygosity; uH_E_, unbiased expected heterozygosity; F, fixation index. Ar and PAr were estimated by rarefaction to 18 gene copies (ADZE). SE was calculated across loci.

**Table 4:** F-statistics (AMOVA) across 16 STR loci in the five horse groups.

Locus	F_IS_	F_IT_	F_ST_	*P* (F_ST_)
AHT4	−0.070	−0.051	0.0175	0.001 *
AHT5	−0.029	−0.026	0.0033	0.180
ASB17	−0.007	0.000	0.0071	0.038
ASB2	0.001	0.007	0.0057	0.041
ASB23	−0.003	0.007	0.0095	0.011
CA425	−0.016	−0.013	0.0024	0.217
HMS1	−0.026	−0.019	0.0068	0.099
HMS2	0.018	0.025	0.0072	0.058
HMS3	0.042	0.041	−0.0011	0.573
HMS6	−0.051	−0.036	0.0149	0.016
HMS7	0.043	0.052	0.0087	0.016
HTG10	−0.023	−0.018	0.0057	0.062
HTG4	0.041	0.082	0.0430	0.001 *
HTG6	−0.031	−0.016	0.0147	0.007
HTG7	−0.041	−0.040	0.0014	0.337
VHL20	−0.006	−0.006	−0.0001	0.408
Mean	−0.0095	−0.0006	0.0088 (95% CI 0.0051–0.0142)	0.001

*P*, permutation test (999 permutations); * significant after Bonferroni correction for 16 loci (*p* < 0.0031). The 95% CI for the multilocus F_ST_ was obtained by bootstrapping over loci (1000 replicates).

**Table 5:** Analysis of molecular variance (AMOVA) and F-statistics, n = 703.

Source of Variation	df	SS	MS	Variance Component	%	F-Statistic	*P*
Among groups	4	42.932	10.733	0.051	0.87	F_ST_ = 0.0088	0.001
Among individuals within groups	698	3997.568	5.727	0.000	0.00	F_IS_ = −0.0095	0.921
Within individuals	703	4103.500	5.837	5.837	99.13	F_IT_ = −0.0006	0.573
Total	1405	8144.000	—	5.889	100	—	—

*P*, permutation test (999 permutations).

**Table 6:** Pairwise F_ST_ (Weir and Cockerham), n = 703.

Pair	F_ST_	95% CI	*P*
CROSS–KST	0.0150	0.0020–0.0297	0.060
CROSS–KT	0.0463	0.0234–0.0757	0.002 *
CROSS–TB	0.0051	−0.0082–0.0194	0.225
CROSS–UB	0.0284	0.0121–0.0489	0.003 *
KST–KT	0.0101	0.0038–0.0181	0.022
KST–TB	0.0076	0.0013–0.0138	0.038
KST–UB	0.0017	−0.0005–0.0040	0.167
KT–TB	0.0289	0.0134–0.0508	0.001 *
KT–UB	0.0083	0.0035–0.0129	0.005
TB–UB	0.0090	0.0023–0.0180	0.002 *

95% CI, confidence interval obtained by bootstrapping over loci (1000 replicates); *P*, permutation test (999 permutations); * significant after Bonferroni correction for 10 pairs (*p* < 0.005).

**Table 7:** Results of the assignment test, n = 703.

Original Group (n)	→ CROSS	→ KST	→ KT	→ TB	→ UB	Correct, %
CROSS (9)	5	1	0	3	0	55.6
KST (32)	4	13	2	5	8	40.6
KT (36)	0	8	18	3	7	50.0
TB (31)	2	9	0	12	8	38.7
UB (595)	15	136	109	143	192	32.3

Note: The arrow (→) indicates the group to which an individual was assigned.

**Table 8:** Pairwise Nei’s genetic distances among the five studied horse groups.

	CROSS	KST	KT	TB	UB
CROSS	0.000				
KST	0.116	0.000			
KT	0.201	0.061	0.000		
TB	0.093	0.057	0.111	0.000	
UB	0.139	0.024	0.040	0.043	0.000

## Data Availability

The data supporting the findings of this study are provided in the article and its Supplementary Materials, including anonymized individual multilocus genotypes with group and sex (Table S14). Additional data are available from the corresponding authors upon reasonable request.

## Supplementary Materials

The following supporting information can be downloaded at: https://www.mdpi.com/article/10.3390/ani16213453/s1, Table S1: Sampling locations of the studied horses with geographic coordinates; Table S2: Allele frequencies by studied groups; Table S3: MICRO-CHECKER results; Table S4: Close relatives removed from the dataset; Table S5: Hardy–Weinberg equilibrium tests for each locus in each group; Table S6: Linkage disequilibrium tests for all pairs of loci in each group; Table S7: Comparison of the results obtained with 17 and 16 loci (n = 765); Table S8: Per-locus diversity parameters (Na, H_O_, H_E_, F) in the studied groups; Table S9: Overall F_ST_ and DAPC results in the 10 balanced subsamples; Table S10: Identifiers of the UB animals included in the 10 balanced subsamples; Table S11: STRUCTURE results for the final dataset (n = 703); Table S12: STRUCTURE results in the 10 balanced subsamples; Table S13: DAPC results for the final dataset (n = 703); Table S14: Anonymized individual multilocus genotypes of the 765 animals with group and sex; Figure S1: BIC values in the DAPC cluster selection; Figure S2: DAPC membership probabilities at *K* = 2–5; ARRIVE 2.0 checklist.
